# GROWTH OF THE PROGRAM OF ALL-INCLUSIVE CARE FOR THE ELDERLY AND THE ROLE OF PRIVATE EQUITY

**DOI:** 10.1093/geroni/igae098.3093

**Published:** 2024-12-31

**Authors:** Katherine Miller, Ravi Gupta, Daniel Polsky

**Affiliations:** Johns Hopkins University, Baltimore, Maryland, United States; Johns Hopkins University School of Medicine, Baltimore, Maryland, United States; Johns Hopkins University, Baltimore, Maryland, United States

## Abstract

The Program of All-Inclusive Care for the Elderly (PACE) is a managed care program for adults 55+ who require nursing home level of care and are mostly dual-eligible for Medicare and Medicaid. PACE programs receive capitated payments per person for comprehensive medical and social services to enrollees, primarily in adult day health centers. In 2016, the Centers for Medicare & Medicaid began allowing for-profit ownership of PACE. Our objective is to describe the growth of PACE from 2010 to 2022 in the context of the 2016 policy change allowing for for-profit investment. We also describe the share of for-profit programs receiving private equity investments. We use Refinitiv and Medicare Advantage contract and enrollment data. Enrollment in for-profit programs from 2016 to 2022 increased by an annual average of 13% compared to 5.6% in non-profit programs. This is the context of an annual rate of increase in enrollment of 11% in the 6 years pre-policy change and 7% in the 6 years post-policy change. Among enrollees at for-profit programs in 2022, 47% received care at programs with private equity investments. Despite the growth of for-profit ownership and private equity investments, most enrollees received care from non-profit programs (78%) in 2022. Since allowing for-profit ownership, expansion has been driven by for-profit entities, ~50% of which received private equity investments. While expanding access to PACE may be important for certain adults, examining the effects of growth in for-profit and private equity ownership of PACE programs for patient outcomes is an important next step.

